# Supplementary material for: Non-classical correlations over 1250 modes between telecom photons and 979-nm photons stored in 171Yb3+:Y2SiO5
Source: Nat Commun. 2022 Oct 28;13:6438. doi: 10.1038/s41467-022-33929-y (PMC9616888; doi:10.1038/s41467-022-33929-y)
Supplement: Supplementary file 1 — Supplementary Information [file 41467_2022_33929_MOESM1_ESM.pdf]

Supplementary Information: "Non-classical correlations over 1250 modes between telecom photons and 979-nm photons stored in $^{171}Yb^{3+}:Y_2SiO_5$"

M. Businger, L. Nicolas, T. Sanchez Mejia, and Mikael Afzelius*
Départment de Physique Appliquée, Université de Genève, 1205 Genève, Switzerland

A. Ferrier
Chimie ParisTech, PSL University, CNRS, Institut de Recherche de Chimie Paris, 75005 Paris, France and Faculté des Sciences et Ingénierie, Sorbonne Université, UFR 933, 75005 Paris, France

P. Goldner
Chimie ParisTech, PSL University, CNRS, Institut de Recherche de Chimie Paris, 75005 Paris, France

* Email to: mikael.afzelius@unige.ch

SUPPLEMENTARY NOTE 1 - PREPARING A BROADBAND AFC WITH MANY COMB LINES

In this section, we discuss the problem of burning a large bandwidth ($\Delta_\nu = 100$ MHz) AFC composed of a high number of teeth ($N > 1000$). A pulse sequence allowing more efficient use of the limited peak optical power is proposed.

The AFC is created using a succession of frequency-chirped pulses, where each chirped pulse optically pumps away atoms in between the AFC comb teeth. The frequency of the pulses are periodically spaced by the desired AFC periodicity Δ. Each pulse is an adiabatic secant hyperbolic (sech) pulse [1, 2] that has a squarish frequency spectrum, provided that the duration of the pulse τ is longer than $1/\Delta_\mathrm{f}$, Δ_f being the chirp range of the sech pulse. The amplitude of the sech pulse that burns tooth n can be written as in Eq. 1,

$$A_n(t) = \mathrm{sech}(\beta t)\sin\left[2\pi(f_0 + n\Delta)t + 2\pi\frac{\Delta_\mathrm{f}}{2\beta}\ln(\cosh(\beta t))\right], \text{with } t \in [-\tau/2, \tau/2]\,. \quad (1)$$

where $n\Delta$ is the relative shift from the central frequency of the AFC f_0. β is a parameter related to the temporal width of the pulse [1, 2], which is determined by τ (for this numerical study, $\beta = 10/\tau$).

In this study, we work with AFCs with a very large number of teeth. Thus, it is not suitable to burn each tooth one after the other as the total preparation time would take far too long. In ref [2], a method to burn all the teeth of the comb simultaneously was proposed, where the pulse amplitudes $A_n(t)$ corresponding to all the teeth are summed. For N comb teeth, this parallel method results in a total pulse amplitude

$$\begin{aligned} A(t) &= \sum_{n=-N/2}^{N/2-1} A_n(t), \\ &= \mathrm{sech}(\beta t)\frac{\sin(N\pi\Delta t)}{\sin(\pi\Delta t)}\cos\left[2\pi f_0 t + 2\pi\frac{\Delta_\mathrm{f}}{2\beta}\ln(\cosh(\beta t))\right]. \end{aligned} \quad (2)$$

When N is high, Eq. 2 results in a pulse train in the time domain where the energy is concentrated in very sharp and intense peaks (cf. the trace of Supplementary Figure 1). Indeed, summed this way, all the chirps interfere destructively except for $\Delta t \in \mathbb{Z}$ when they interfere constructively. As the teeth do not overlap in frequency, the available energy for each tooth is not affected by the sum. In other words, $E_\mathrm{comb} = NE_0$, where $E_\mathrm{comb} = \int_{-\tau/2}^{\tau/2}|A(t)|^2\,dt$ is the total energy of $A(t)$ and $E_0 = \int_{-\tau/2}^{\tau/2}|A_n(t)|^2\,dt$ is the pulse energy of each pulse $A_n(t)$.

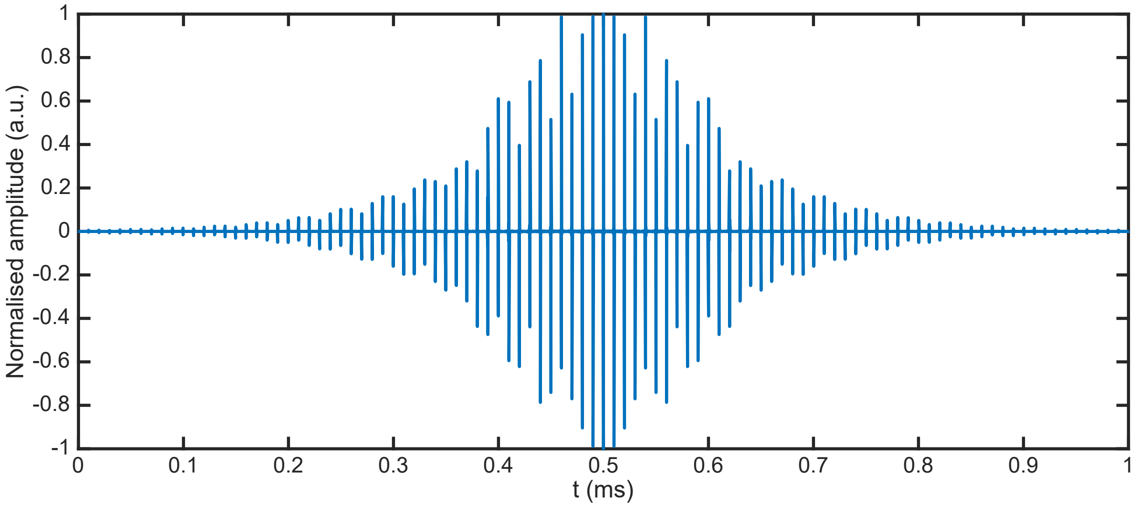

Supplementary Figure 1: Temporal profile of the pulse using the regular parallel method. $N = 1000$ and $\Delta_\nu = 100$ MHz. $\tau = 1$ ms, $\beta = 10/\tau$ and $t \leftarrow t + \tau/2$.

In general, the amplitude after the modulator, $A_\mathrm{EOM}(t)$, has a limited peak amplitude. This is true independently of the type of modulator, acousto-optic modulators (AOMs) or electro-optic modulators (EOMs). The maximum

amplitude is limited by the input laser amplitude, insertion losses, maximum modulation depth etc.. In the case of an EOM, one should also avoid overmodulation to reduce possible distortions. We denote m as the maximum amplitude of the modulated signal $A_{\mathrm{EOM}}(t)$. A first order expansion reveals that the factor $\sin(N\pi\Delta t)/\sin(\pi\Delta t) = \pm N$ when $\Delta t \in \mathbb{Z}$. To exploit the full range of the EOM, we can use the signal $A_{\mathrm{EOM}}(t) = m\frac{A(t)}{N}$. The total energy in the pulse is then

$$E_{\mathrm{EOM}} = \int_{-\tau/2}^{\tau/2} \left| m\frac{A(t)}{N} \right|^2 dt = \frac{m^2}{N^2} E_{\mathrm{comb}} = \frac{m^2}{N^2} N E_0 = \frac{m^2}{N} E_0. \tag{3}$$

We observe that the limited EOM output amplitude results in loss in pulse energy as the number of teeth is increased, as $E_{\mathrm{EOM}} \propto 1/N$. In Supplementary Figure 2(a), the numerically-processed and normalised energy $E_{norm} = \frac{E_{\mathrm{EOM}}}{E_0 m^2}$ is plotted, confirming the $1/N$ decay of the energy as N is increased. In the following, we will compare numerically computed pulse trains, which we normalize with respect to the maximum output amplitude m, such that the total pulse energy can be computed by

$$E_{\mathrm{EOM}} = \int_{-\tau/2}^{\tau/2} \left| m\frac{A(t)}{\mathbf{max}\left(|A(t)|\right)} \right|^2 dt. \tag{4}$$

The loss of pulse energy as N is increased posed a serious problem in the current work, where N reached up to $N = 2500$, which spurred us to find a solution to the problem. In fact, in the field of telecommunications, this loss of available energy due to interferences in the time domain is a known problem (cf. [3, 4]). A solution to this problem, proposed in [3] by Shroeder, is to add a phase Φ_n to each pulse n before the sum

$$\Phi_n = \pi\mathbf{round}\left(\frac{n^2}{2N}\right). \tag{5}$$

As a result, the signal does not display sharp peaks and the energy is distributed over all the pulse length while still maintaining a hyperbolic secant envelope. After having normalized the pulse by its maximum value, cf. Eq. 4, we observe that the pulse energy does not depend on N. The normalized energy is plotted in green in Supplementary Figure 2(a). In Supplementary Figure 2(b), this energy divided by the normalized pulse energy using the parallel method is plotted. For large N, the gain in energy is significant.

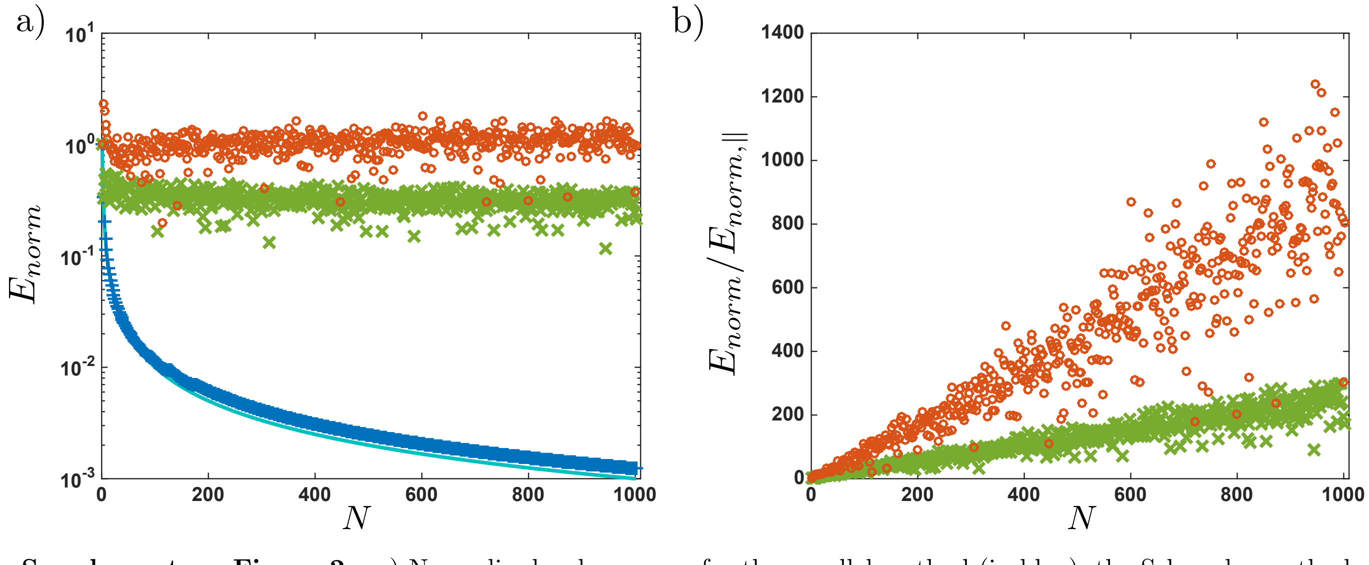

Supplementary Figure 2: a) Normalised pulse energy for the parallel method (in blue), the Schroeder method (in green) and our new method (in red) as a function of the number of teeth N. The blue line shows the $1/N$ dependence. b) Pulse energy for the Schroeder method (in green) and our new method (in red) divided by the pulse energy for the parallel method, as a function of the number of teeth N.

In this work, we propose a slightly different solution, which results in an even higher pulse energy for the same number of comb lines N. Instead of summing up all the N pulses simultaneously, as in Eq. 2, each sech pulse

$A_n(t)$ starts at a slightly different time. To do so numerically, we apply a circular permutation of the time vectors corresponding to the pulses before summing them and normalizing by the maximum value of the obtained signal. The construction of such a pulse is described in Supplementary Figure 3(a) and (b). Eq. 6 gives an analytical description of the total AFC preparation pulse $A(t)$,

$$A(t) = \sum_{n=-N/2}^{N/2-1} \begin{cases} A_n\left(t+\tau-(n+\frac{N}{2}+1)\frac{\tau}{N}\right) & \text{if } t \in \left[-\frac{\tau}{2}, (n+\frac{N}{2}+1)\frac{\tau}{N}-\frac{\tau}{2}\right], \\ \\ A_n\left(t-(n+\frac{N}{2}+1)\frac{\tau}{N}\right) & \text{if } t \in \left](n+\frac{N}{2}+1)\frac{\tau}{N}-\frac{\tau}{2}, \frac{\tau}{2}\right]. \end{cases} \tag{6}$$

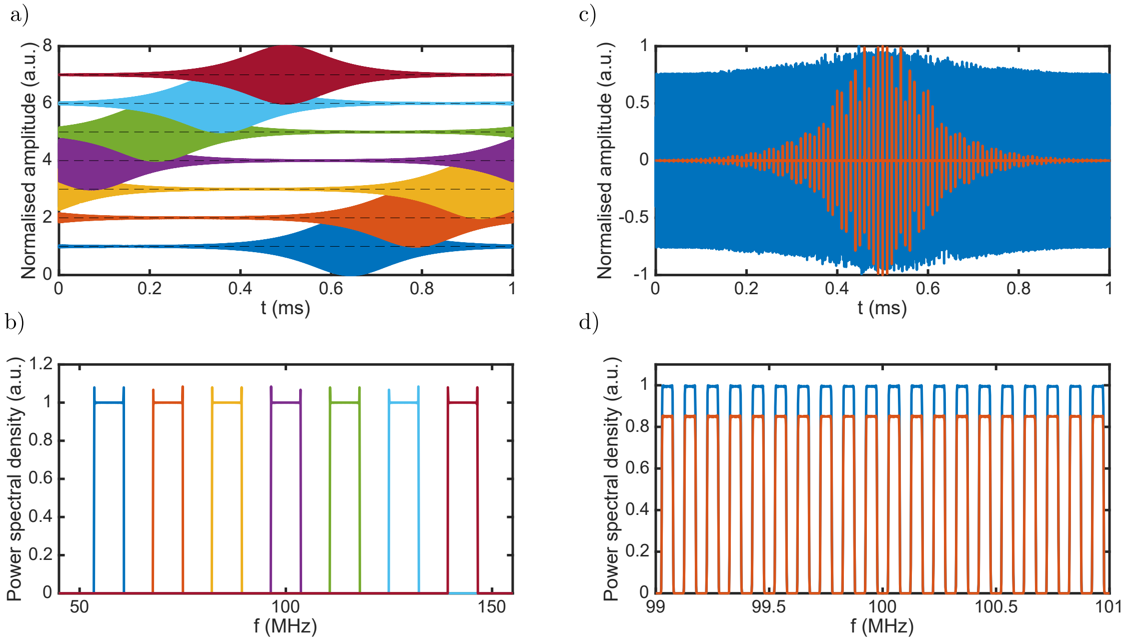

Supplementary Figure 3: a) Seven time-shifted chirp pulses used to create the AFC burning pulse. They are summed and the resulting signal is normalised. b) Power spectrum of the pulses plotted in a). The spectrum of the resulting pulse is the sum of the plotted spectra. c) In red, temporal profil of the pulse using the regular parallel method. $N = 1000$ and $\Delta_\nu = 100$ MHz. In blue, temporal profil of the pulse resulting from the new method. d) Fourier spectra of the two temporal profiles below. The one corresponding to the regular method (in red) is multiplied by 1000. For all these figures, $\tau = 1$ ms, $\beta = 10/\tau$ and $t \leftarrow t + \tau/2$.

With this pulse construction, the energy is homogeneously distributed over the entire pulse duration, without the hyperbolic secant envelope, see Supplementary Figure 3(c). This explains why the total energy of our pulse is higher than when using the Shroeder method, as shown in Supplementary Figure 2(a). In Supplementary Figure 2(b), we plot the pulse energy of the new pulse and of the pulse based on the Schroeder method, divided by the pulse energy based on the parallel method of Ref. [2]. One can see that the energy gain of both pulses is very significant and that it scales linearly with N. The gain is even higher for our method, as compared to the Schroeder method, and the gain approaches N. In Supplementary Figure 3(c), the power density spectra of the two signals are plotted. They both present well-resolved teeth ($N = 1000$) over a broad frequency range ($\Delta_\nu = 100$ MHz).

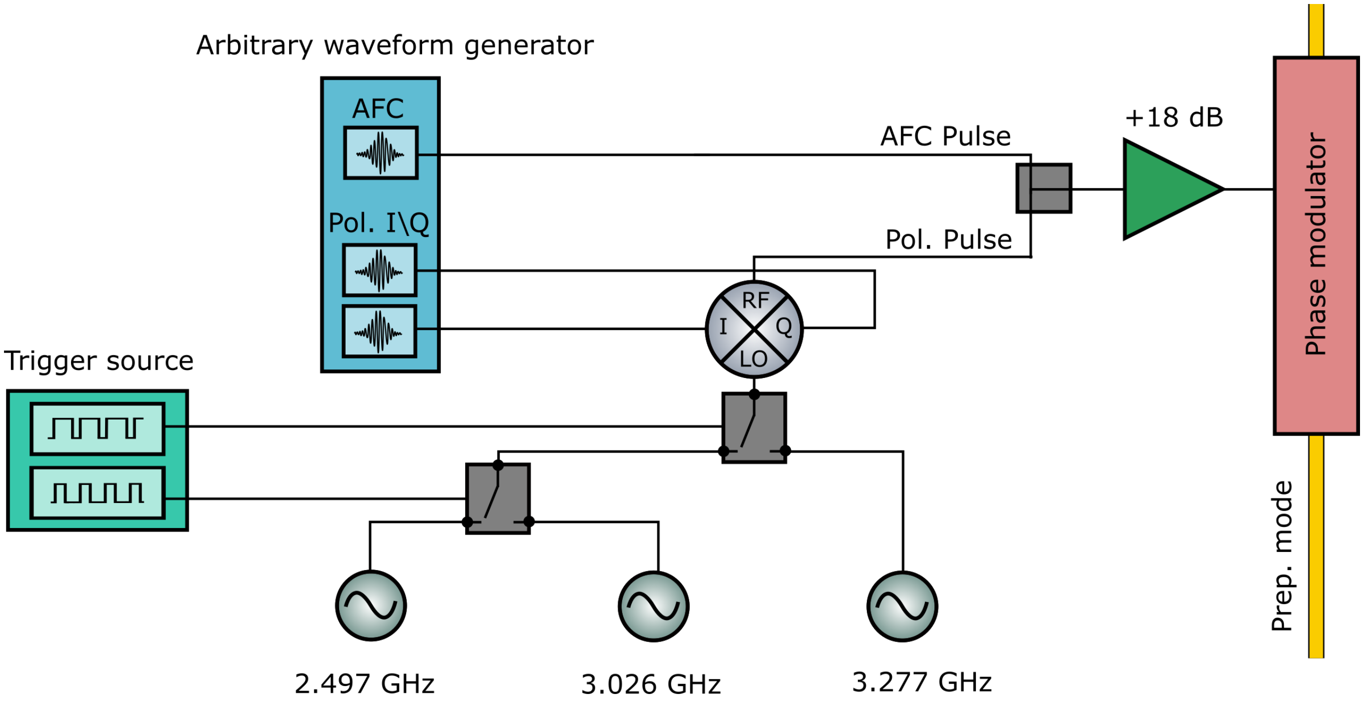

Supplementary Figure 4: Schematic representation of the microwave setup for producing the preparation pulses and driving the phase modulator, as discussed in detail in Supplementary Note 1.

SUPPLEMENTARY NOTE 2 - ELECTRONIC SETUP

The full electronic setup to generate the spin polarization and AFC preparation pulses is depicted in Supplementary Figure 4. To produce the complex amplitude and phase modulation pulses that are GHz apart, we divide the generation in two branches. In the first branch, the AFC preparation pulses (see Supplementary Note 1) are generated by an arbitrary waveform generator (Signadyne, SD AWG-H3344) with a central frequency of 150 MHz and a bandwidth of 100 MHz. These pulses are sent directly to the EOM phase modulator since the laser is already locked on the (1) transition. In the second branch, the spin polarization pulses are generated by upconverting the AWG output with an external IQ-mixer (Marki, IQ-1545). The AWG generates hyperbolic secant pulses that also have a central frequency of 150 MHz with a 100 MHz chirp, which are upconverted to several GHz through the IQ-mixer. To suppress one of the sidebands of the upconversion process, the AWG generates two signals (the I and Q signals), with a relative phase difference of $\pi/2$. This supresses the lower sideband after the IQ mixer. To address all three spin polarization frequencies (2)-(4), we drive the local oscillator of the IQ-mixer with three RF-sources at 2.497 GHz, 3.026 GHz and 3.277 GHz. We switch between them using RF-switches that are driven with an external trigger source, which produces gating pulses that are synchronized with the AWG pulses. The upconverted spin polarization pulses are then combined with the AFC pulse branch using a passive RF combiner, amplified with a broadband amplifier (SHF Communication Technologies, SHF-100-CP) and then sent to an EOM phase modulator (Eospace, PM-0S5-PFA-PFA-880) to generate the optical preparation pulses.

SUPPLEMENTARY NOTE 3 - AUTO-CORRELATION FUNCTIONS

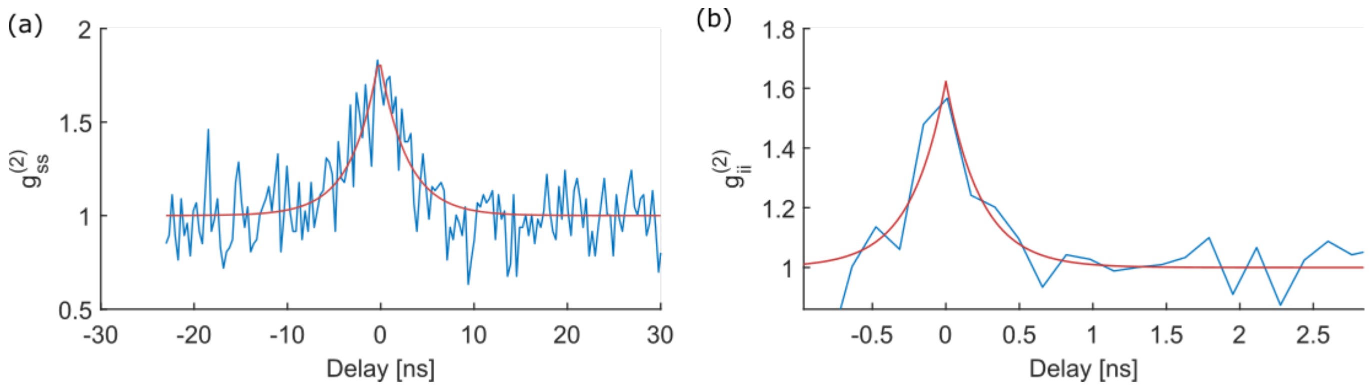

Supplementary Figure 5: Measurement of the second-order auto-correlation function of the signal photons at 979 nm in (a) and the idler photons at 1550 nm in (b). For the signal (idler) photons, we used a binning of 320 ns (160 ns).

The individual modes of a two-mode squeezed vacuum state obey thermal statistics, hence have second-order auto-correlation functions of $g^{(2)}_{kk} = 2$ $(k = i, s)$. An SPDC source filtered down to a single idler and signal mode, respectively, should ideally obey the same photon statistics. In this note, we present measurements of the second-order auto-correlation functions of the signal and idler photons, but without the memory to avoid the losses on the 979 nm mode, shown in Supplementary Figure 5. We find a peak auto-correlation of $g^{(2)}_{ss}(0) = 1.8$ for the signal photons and $g^{(2)}_{ii}(0) = 1.6$ for the idler photons. We also fit the data using the formula from Ref. [5] and extract the linewidth of 660(200) MHz for the idler photons and 54(15) MHz for the signal photons. The peak value for the idler photons is reduced by the jitter of the detection system. For the signal photon, the jitter is negligible, and from the reduced value of 1.8 we extract a Schmidt mode number of K = 1.2(2) [5]. This slightly higher mode number could indicate that the FP cavity also transmits other modes through the VBG, which could be due to coupling into weak transverse modes in the FP cavity.

SUPPLEMENTARY NOTE 4 - EFFICIENCY DECAY

In this note, we present measurements of the AFC memory efficiency using bright coherent states detected by a photodiode, as a function of the AFC memory delay $1/\Delta$, see Supplementary Figure 6. The input pulse had a duration of 1 μs, with its carrier frequency centered on the AFC spectrum. The decay curve was fitted to the formula $\eta = \eta_0 \exp\big(-4/(\Delta T_2^{\mathrm{AFC}})\big)$ [2], resulting in $T_2^{\mathrm{AFC}} = 69(6)$ μs and a zero-delay efficiency of $\eta_0 = 12(2)\%$. The error bars corresponds to 95% confidence intervals.

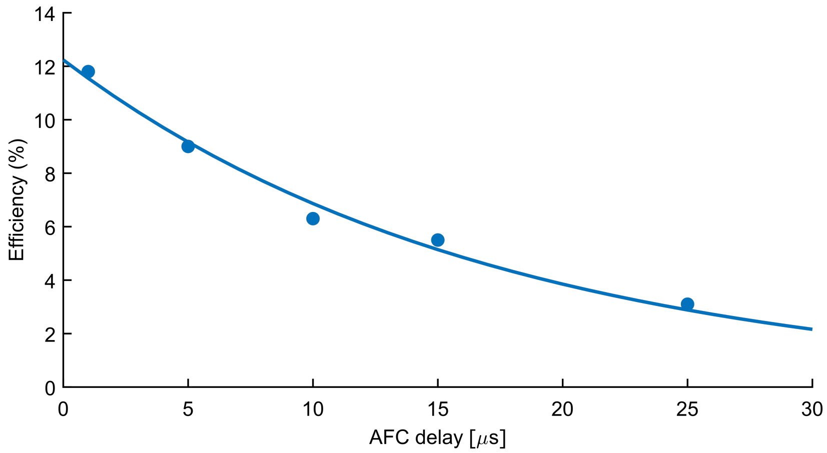

Supplementary Figure 6: AFC memory efficiency as a function of the storage time $1/\Delta$, measured with bright laser pulses.

SUPPLEMENTARY NOTE 5 - POWER STUDY OF PHOTON DETECTION RATES AND THE SECOND-ORDER CROSS-CORRELATION FUNCTION

In this note, we present a short power study of the single photon detection rates and second-order cross-correlation function $g_{si}^{(2)}$. These are measured while detecting either the 979 nm photons transmitted through the memory (not absorbed by the memory), or the 979 nm photons stored and later emitted in the AFC echo, for a fixed storage time of $1/\Delta = 1$ μs. The power of the 600 nm pump light is varied between 130 μW to 270 μW, using a polarizing beam splitter and a half-wave plate in front of the SPDC source. The maximal power of the 600 nm light was limited by the injected 979 nm pump power of the SFG setup.

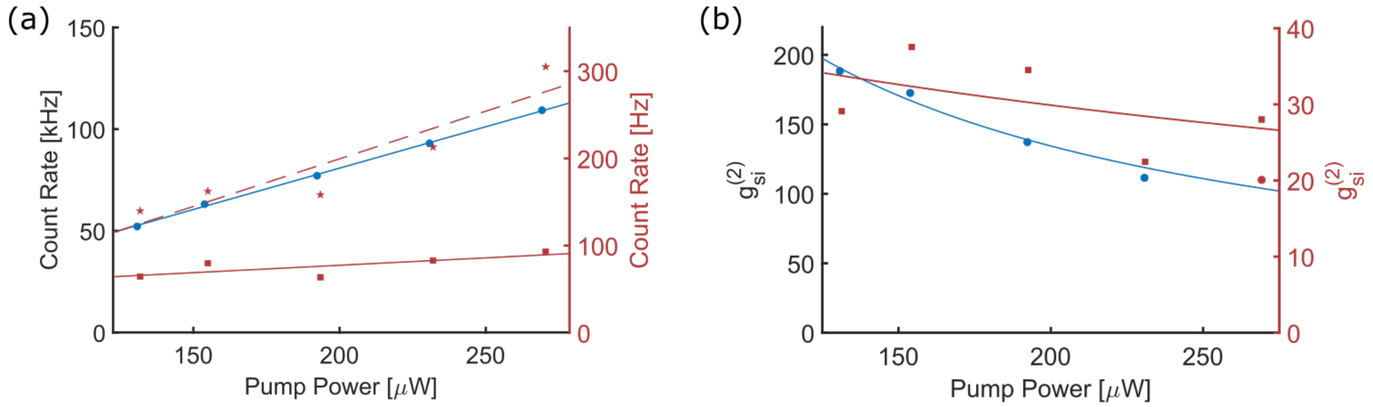

Supplementary Figure 7: (a) Count rates for the idler photons at 1550 nm (blue, left axis), and transmitted (red squares, right axis) and stored (red stars, right axis) signal photons at 979 nm, as a function of SPDC pump power. The storage time was set to $1/\Delta = 1$ μs. (b) Peak cross correlation function $g_{si}^{(2)}(0)$ between the idler photons and signal photons in the transmission window (blue, left axis) and echo window (red squares, right axis).

The photon detection rates are shown in Supplementary Figure 7, for both the transmitted and stored 979 nm photons (signal photons), and the 1550 nm photons (idler photons). All count rates follow the expected linear dependence on the pump power. The count rate of the heralding telecom photons is generally high, between 50 and 100 kHz over this range of power. It should be noted that the heralding rate within the effective bandwidth of the stored signal photons is lower, due to the highly asymmetric filtering. Still, taking into account the 500 MHz idler filter and the 64 MHz signal filter, we obtain a high estimated heralding rate of about 6 kHz for the idler photons. On the other hand, the count rate of the stored AFC echo signal is below 100 Hz over the entire range. This is due to the limited memory efficiency, low detector efficiency, the insertion loss of the narrowband FP cavity, and losses due to the four-pass configuration through the cryostat (crystal surfaces were not AR coated). Less losses and a more efficient detector would significantly increase the signal rate, and hence also the coincidence rate. It would also increase the second-order cross-correlation function $g_{si}^{(2)}(0)$ of the stored photons, as the 979 nm detection rate is not significantly larger than the dark count rate of about 30 Hz.

In Supplementary Figure 7, the measured peak second-order cross-correlation value $g_{si}^{(2)}(0)$ is plotted as a function of the pump power P_{p}, again for both the transmitted and stored 979 nm photons. The $g_{si}^{(2)}(0)$ follows the expected $1/P_{\mathrm{p}}$ dependence when detecting the transmitted signal photons, but much less clearly when detecting the stored signal photons. This is mostly due to the low count rate, and possibly due to fluctuations in the memory efficiency during the experiment. Nevertheless, the data is in general agreement with the expected behavior of a two-mode squeezed vacuum state, as ideally produced by a SPDC source, for low pump powers.

SUPPLEMENTARY REFERENCES

[1] L. Rippe, M. Nilsson, S. Kröll, R. Klieber, and D. Suter, Phys. Rev. A **71**, 062328 (2005).
[2] P. Jobez, N. Timoney, C. Laplane, J. Etesse, A. Ferrier, P. Goldner, N. Gisin, and M. Afzelius, Phys. Rev. A **93**, 032327 (2016).
[3] M. Schroeder, IEEE Transactions on Information Theory **16**, 85 (1970).
[4] R. Oswald, A. Y. Nevsky, and S. Schiller, Physical Review A **104**, 063111 (2021).
[5] C. Clausen, F. Bussières, A. Tiranov, H. Herrmann, C. Silberhorn, W. Sohler, M. Afzelius, and N. Gisin, New Journal of Physics **16**, 093058 (2014).
